# Mitochondrialand: What Will Come Next?

**DOI:** 10.1093/function/zqab073

**Published:** 2021-12-27

**Authors:** Paola Pizzo, Tullio Pozzan

**Affiliations:** Department of Biomedical Sciences, University of Padua, 35131 Padua, Italy; Neuroscience Institute, National Research Council (CNR), 35131 Padua, Italy

The interest of the scientific community towards mitochondria and the multiple roles of this organelle in physiology and pathology has enormously increased in the last two decades. Such an increase depends on different factors, from the discovery of the role of mitochondria in signaling to their control of cell death and from their importance in metabolism to the discovery of their crosstalk with other organelles. In our (surely biased) opinion, the major breakthrough in the field of mitochondrial functions of the last decade concerns the identification of the molecular components of the Ca^2+^ handling mechanisms, *i.e*., the Na/Ca^2+^ exchanger (NCXL) and the “Ca^2+^ uniporter complex”. In addition, the description of a still partially undefined molecular machinery that regulates the interaction of mitochondria with the other organelles is also of major relevance.

In the last year, although the field has not seen dramatic breakthroughs, we have identified three aspects of mitochondrial physiopathology where important novel findings have been obtained that we expect could represent the topic for further investigations in the near future: (i) The causal role of mitochondrial dysfunction in neurodegeneration; (ii) mitochondrial interorganelle contacts and functional coupling; and (iii) mitochondria transfer between different cells and organs.

## The Causal Role of Mitochondrial Dysfunction in Neurodegeneration

Evidence supporting a role of mitochondrial dysfunctions in several neurodegenerative pathologies, eg Alzheimer's disease, ataxia telangectasia, and amyotrophic lateral sclerosis have been obtained in the last decade. However, the one that most deserves the classification of “mitochondrial disease” is surely Parkinson's disease (PD). Although its pathogenesis is multifactorial, several gene mutations causing early-onset PD (as well as some environmental factors increasing PD risk) affect the function of proteins involved in mitochondrial activity. However, while for a few mutations in mitochondrial proteins the causal relationship between PD and organelle dysfunctions is undisputed, it is debated whether mitochondrial defects described in sporadic PD represent a cause or a consequence of the neurodegenerative pathways.

In a recent paper, Gonzàlez-Rodriguez *et al*.^1^ demonstrate that, in mice, the direct disruption of mitochondrial complex I in dopaminergic neurons (by conditional *Ndufz2* knock out) is sufficient to induce progressive dysfunction and degeneration of these cells, similarly to what observed in human parkinsonism. In particular, the initial defects are restricted to nigrostriatal axons leading to loss of striatal dopamine release and fine motor deficits. Then, neuronal bodies and dendrites also degenerate, impacting on dopamine release in the *substantia nigra*. Only at this later stage, gross motor deficits appear. These results indicate that mitochondrial health is crucial for dopaminergic neuron life and, importantly, suggest that there is a time window from the first symptoms in which possible therapeutic approaches could be developed to prevent/delay complete neuronal degeneration and the appearance of overt motor defects.

Future research will show us whether mitochondrial activity is indeed essential also for other types of neurons to become the principal target of diverse neurodegenerative disease-modifying therapies.

## Mitochondrial Inter-Organelle Contacts and Functional Coupling

Mitochondria–endoplasmic reticulum (ER) close appositions have been noticed since the 70’s, but for a long time they were considered electron microscopy artifacts. At the beginning of the 90’s it was demonstrated that these contacts are responsible for both the generation of high Ca^2+^ microdomains, allowing mitochondria to rapidly accumulate Ca^2+^ upon its release from the ER and the coordinated synthesis and transport of some lipids at the organelle interface (biochemically isolated and named MAM, mitochondrial associated membranes). The search for proteins involved in these structures was a major topic in the last two decades (with, in some cases, strongly debated results).

Although mitochondria make functional contacts not only with the ER but with almost all the other organelles, modulating a wide variety of cell functions (often dysregulated in many diseases), the functional coupling between the nucleus and mitochondria has not been deeply investigated.

Very recently, Eisenberg-Bord *et al*.^2^ identified, in *Saccharomyces cerevisiae*, the uncharacterized protein Ybr063c (named Cnm1, contact nucleus mitochondria 1) as the tethering molecule between mitochondria and the nuclear membrane. Cnm1 is an integral nuclear membrane protein which needs the outer mitochondrial membrane protein Tom70 to tether mitochondria to the nucleus. These interorganelle contacts in yeast are functionally regulated by phosphatidylcholine levels, thus coupling (together with another multimolecular complex at the mitochondria–ER interface, ERMES)^3^ phospholipid homeostasis between the two organelles with the modulation of the contact length. It is not known whether a similar homeostatic process is present in mammalian cells, with the involvement of a Cnm1 homologue and Tom70 (indeed, an additional function for this protein, independent from the TOM complex for protein import, has been already described).^4^ Along the same line, novel functional interorganelle communications have been recently described in mammals, sustaining a stress-induced, mitochondria-to-nucleus retrograde signaling response to activate pro-survival programs.^5,6^

We foresee a big expansion of mitochondrial “contactology” in the next years, focused in analysing the dynamic nature of these sites, their regulation in physio-pathological conditions, and the molecules involved in their formation.

## Mitochondria Transfer Between Different Cells and Organs

In the last decade, several studies have pointed out that mitochondria can be transferred from one type of cell to another via specific pathways, mainly tunneling nanotubules (TNT) and extracellular vesicles (EVs). The functional role of this mitochondrial transfer could be multiple, depending on the cells involved and the stimulus triggering the process. For instance, a Miro-1 dependent TNT-mediated transfer of mitochondria from mesenchymal stem cells (MSCs) to bronchial epithelial cells has been reported to be protective in different pulmonary disease mouse models,^7^ eg enhancing cell bioenergetics, ameliorating acute lung injury, and improving survival. In addition, oligodendrocytes transfer material to be degraded to microglia^8^ via EVs and MScs are able to deliver mitochondrial-EVs to cells lacking mtDNA, rescuing their metabolic activity.^9^

Very recently, Crewe *et al*.^10^ showed that an EV-mediated mitochondrial transfer could occur between different tissues, producing a sort of inter-organ “mitohormesis”. They demonstrated that obese mice have higher plasma levels of EVs containing oxidatively damaged mitochondria, which are released by dysfunctional stressed adipocytes and incorporated into the cardiac mitochondrial network, triggering an oxidative burst. This, in turn, activates a compensatory antioxidant program which limits cardiac ischemia/reperfusion injury in these mice. Importantly, obese, metabolically impaired people have been shown to have more circulating mitochondrial-containing EVs compared to controls, suggesting that the process may have a key role also in humans and explaining why these patients have a higher incident of cardiovascular disease but a better prognosis than lean individuals.

It will be interesting to explore the possibility that mitochondria-containing EVs, and not only soluble cytokines, represent a common danger-signal in different disease-associated inter-organ communications to become a suitable target for pharmacological intervention.

## Funding

The authors acknowledge funding by the University of Padova, Italy (SID 2019) and the Italian Ministry of University and Scientific Research (PRIN2017XA5J5N) to P.P., the UNIPD Funds for Research Equipment (2015), Fondazione Cassa di Risparmio di Padova e Rovigo (CARIPARO Foundation) Excellence project 2017 (2018/113), and the Euro Bioimaging Project Roadmap/ESFRI from the Ministry of University and Research of Italy to T.P.
